# 7-day weighed food diaries suggest patients with hereditary hemorrhagic telangiectasia may spontaneously modify their diet to avoid nosebleed precipitants

**DOI:** 10.1186/s13023-017-0576-6

**Published:** 2017-03-28

**Authors:** Helen Finnamore, B. Maneesha Silva, B. Mary Hickson, Kevin Whelan, Claire L. Shovlin

**Affiliations:** 1 National Heart and Lung Institute Cardiovascular Sciences, Imperial College London, London, UK; 2 Imperial College School of Medicine, Imperial College London, London, UK; 3 Diabetes and Nutritional Sciences Division, King’s College London, London, UK; 4 Nutrition and Dietetics, Plymouth University, Plymouth, UK; 5 Respiratory Medicine, Imperial College Healthcare NHS Trust, London, UK

**Keywords:** Anemia, Epistaxis, Fish oil, Hemorrhage adjusted iron requirement (HAIR), Iron, 7-day weighed food diary

## Abstract

Hereditary hemorrhagic telangiectasia (HHT) poses substantial burdens due to nosebleeds and iron deficiency resulting from recurrent hemorrhagic iron losses. Recent studies by our group found surprising links between HHT nosebleeds and certain food groups. In this letter, we report 7-day weighed food diary assessments of an unselected group of 25 UK patients with HHT whose nosebleeds ranged from mild to severe (median epistaxis severity score 4.66, range 0.89– 9.11). The diaries provide evidence that food items most commonly reported to provoke nosebleeds were ingested by fewer HHT patients, compared to food items less commonly reported to provoke nosebleeds (chi-squared *p* <0.001).

## Introduction

Nosebleeds (epistaxis) occur very frequently in patients with hereditary hemorrhagic telangiectasia (HHT) and significantly impact on quality of life. Typically, nosebleeds severity varies over time, and nosebleeds often come in clusters. Most HHT patients experience nosebleeds at least once a week, and in many series, more than a third experience them on a daily or near-daily basis [1, 2]. In one survey, 326/666 (49%) of unselected respondents with HHT had required specialist invasive treatments, often requiring multi-modality therapy [1]. Through under-replacement of hemorrhagic iron losses, nosebleeds commonly result in iron deficiency anemia, regular use of iron tablets, and in as many as 30% of cases, multiple iron infusions or blood transfusions [3].

Nosebleeds result from the presence of abnormal nasal vasculature, and are the usual primary outcome measure in clinical trials of new HHT therapeutic agents. In two large international surveys by our group, multiple dietary items were reported to exacerbate HHT nosebleeds [1, 2]. Here we report data that suggests some HHT patients may spontaneously modify their diet to avoid food items perceived to provoke nosebleeds.

## Methods and results

In 2011, with ethical approval from the London Wandsworth Research Ethics Committee (11/H0803/8), and written informed consent obtained from all participants, an unselected group of 25 HHT patients measured their food intake using a 7-day weighed food diary [4]. Participants were encouraged to follow their normal diet, recording the time, the food or drink consumed, a description of the brand name, and the method of preparation, in addition to the weighed amount in grams, for each different item consumed. Nosebleeds were quantified using the epistaxis severity score which has a maximum score of 10 [5].

From 2012, with ethical approval from the NRES Committee East Midlands-Derby 1 Research Ethics Committee, and online informed consent obtained from all participants, we performed unbiased evaluations of nosebleeds by surveying HHT patients [1, 2]. Following spontaneous reports of dietary nosebleed precipitants [1], our subsequent survey [2] directed participants to 18 different food groups where they were offered tick boxes of i)“This is not part of my diet”, ii)“I have not noticed any difference”, iii)”Seem to bring on nosebleeds”, iv)“Seem to help nosebleeds**”**. In total, 37/265 (14.0%) participants reported that chocolate seemed to bring on their nosebleeds, with strawberries (25/260, 9.6%) and citrus fruits (21/262, 8.0%) the next most commonly reported food groups (Table 1). These findings mirrored those of the earlier survey [1].

**Table 1:** Details of the 1,523 portions of food ingested by the 25 food diary study participants, and reports of nosebleed effects in the 2013 HHT Survey

	2011 7-day weighed food diary			2013 HHT Survey on Nosebleeds				
				Total reports	Food item seemed to bring on nosebleeds		Food item seemed to help nosebleeds	
Category	Number ingesting	Total Portions	Tertile of ingestion	Number ingesting	Number reporting	%	Number reporting	%
Sweets	6	21	1	259	18	6.95	0	0.00
Strawberries/other berries	10	24	1	260	25	9.62	5	1.92
Beans and lentils	11	16	1	258	2	0.78	4	1.55
Savory biscuits	13	21	1	238	5	2.10	0	0.00
Citrus fruits	13	48	1	262	21	8.02	2	0.76
Chocolate	14	29	1	265	37	13.96	1	0.38
Crisps	15	45	2	242	2	0.83	0	0.00
Bananas, melons	16	56	2	260	9	3.46	3	1.15
Sweet biscuits	19	59	2	243	7	2.88	0	0.00
Fast or frozen foods	21	57	2	240	9	3.75	2	0.83
Breakfast cereals	22	126	2	253	5	1.98	0	0.00
Cheese	22	66	2	263	17	6.46	0	0.00
Meat or fish	24	148	3	259	13	5.02	6	2.32
Green vegetables	24	122	3	268	6	2.24	10	3.73
Other vegetables	24	153	3	264	10	3.79	4	1.52
Bread	25	166	3	260	7	2.69	1	0.38
Potatoes, rice and pasta	25	84	3	262	5	1.91	0	0.00
Milk/yoghurt/butter	25	179	3	262	11	4.20	3	1.15

In the 7-day weighed food diary cohort, the epistaxis severity score ranged from 0.89 to 9.11 (median 4.66). When we evaluated the food items ingested by the 25 participants, we noted that the least frequently ingested foods comprised beans/lentils, chocolate, citrus fruits, savory biscuits, strawberries/other berries, and sweets (Table 1). Thus, the food items most commonly reported to precipitate nosebleeds by the HHT Survey respondents (chocolate, strawberries and citrus fruits) were ingested by fewer HHT-affected participants in the weighed food diary assessment (Table 1).

For statistical analyses, all of the foods in the respective tertile were pooled into least consumed, mid- and most consumed foods across all study participants. The least ingested tertile foods were reported to precipitate nosebleeds in 108/1542 cases (7.0%) compared to 49/1501 (3.2%) for the mid tertile and 52/1575 (3.3%) for the most ingested foods (*p* <0.001 by chi-squared test.)

## Conclusion

This is clearly a small study that should be repeated in larger HHT cohorts, and is additionally unable to address potential impacts of portions sizes, food-nosebleed intervals, and other secondary questions that will need to be foci of future studies. Nonetheless, the current study does appear to provide evidence that HHT patients may modify their diet to avoid food items perceived to provoke nosebleeds. We recognise that diet is an important component of people’s lifestyle, and that suggesting restrictions may not be acceptable to many people with HHT. Dietary modification should be a matter for patient choice, and not over-emphasised by clinicians, unless this negatively impacts on nutrient intake. However if nosebleeds have significant impact on patients’ lifestyle and general health then there is an argument that nosebleed-related dietary advice could be part of clinical management. The current observations may offer acceptable opportunities for some people to better control nosebleeds, in addition to improving future HHT nosebleed clinical trial design.

## Abbreviations

ESS: Epistaxis severity score
HAIR: Hemorrhage adjusted iron requirement
HHT: Hereditary hemorrhagic telangiectasia
